# Over-Expression of miR-106b Promotes Cell Migration and Metastasis in Hepatocellular Carcinoma by Activating Epithelial-Mesenchymal Transition Process

**DOI:** 10.1371/journal.pone.0057882

**Published:** 2013-03-06

**Authors:** Wing Lung Yau, Colin Siu Chi Lam, Lui Ng, Ariel Ka Man Chow, Sylvia Tsz Ching Chan, Jacky Yu Ki Chan, Jana Yim Hung Wo, Kevin Tak Pan Ng, Kwan Man, Ronnie Tung Ping Poon, Roberta Wen Chi Pang

**Affiliations:** 1 Department of Surgery, Li Ka Shing Faculty of Medicine, The University of Hong Kong, Hong Kong SAR, China; 2 Center for Cancer Research, Li Ka Shing Faculty of Medicine, The University of Hong Kong, Hong Kong SAR, China; MOE Key Laboratory of Environment and Health, School of Public Health, Tongji Medical College, Huazhong University of Science and Technology, China

## Abstract

Hepatocellular carcinoma (HCC) is one the the most fatal cancers worldwide. The poor prognosis of HCC is mainly due to the developement of distance metastasis. To investigate the mechanism of metastasis in HCC, an orthotopic HCC metastasis animal model was established. Two sets of primary liver tumor cell lines and corresponding lung metastasis cell lines were generated. *In vitro* functional analysis demonstrated that the metastatic cell line had higher invasion and migration ability when compared with the primary liver tumor cell line. These cell lines were subjected to microRNA (miRNAs) microarray analysis to identify differentially expressed miRNAs which were associated with the developement of metastasis *in vivo*. Fifteen human miRNAs, including miR-106b, were differentially expressed in 2 metastatic cell lines compared with the primary tumor cell lines. The clinical significance of miR-106b in 99 HCC clinical samples was studied. The results demonstrated that miR-106b was over-expressed in HCC tumor tissue compared with adjacent non-tumor tissue (p = 0.0005), and overexpression of miR-106b was signficantly correlated with higher tumor grade (p = 0.018). Further functional studies demonstrated that miR-106b could promote cell migration and stress fiber formation by over-expressing RhoGTPases, RhoA and RhoC. *In vivo* functional studies also showed that over-expression of miR-106b promoted HCC metastasis. These effects were related to the activation of the epithelial-mesenchymal transition (EMT) process. Our results suggested that miR-106b expression contributed to HCC metastasis by activating the EMT process promoting cell migration *in vitro* and metastasis *in vivo*.

## Introduction

Hepatocellular carcinoma (HCC) is one of the most common cancers worldwide. It is the third leading cause of cancer deaths worldwide [1]. The incidence of HCC is particularly high in Chinese population due to endemic Hepatitis B virus infection [2]. Liver resection and liver transplantation are curative treatments for early-stage HCC. However, HCC is usually diagnosed at advanced stage and treatment options for metastatic disease are very limited. Molecular pathological studies demonstrated that gene expression alteration is crucial for the development of HCC metastasis.

MicroRNAs (miRNAs) are small non-coding RNA molecules which possess regulatory functions for gene expression. Development of metastasis is associated with alteration of gene expression, hence, miRNAs are suspected to be associated with the development of cancer metastasis. Previous studies have demonstrated that miRNAs play a critical role in development of cancer and metastasis. miR-21 and miR-122a can regulate important tumor suppressors, PTEN and CyclinG1 in HCC [3], [4]. MiRNAs also reported to be associated with metastasis. miR-373 and miR-520c have been reported to be associated with tumor invasion and metastasis in breast cancer cell lines [5]. A unique 20-miRNAs metastasis signature has been identified in HCC clinical samples [6]. These studies demonstrated miRNAs play a critical role not only in development of cancer but also in metastasis. miR-106b is transcripted from the miR-106b-25 cluster located on chromosome 7. This miRNA cluster is reported to be over-expressed in HCC clinical samples [7]. A microarray study demonstrated that miR-106b was up-regulated in colon cancer with lymph node metastasis [8]. miR-106b can directly bind to p21 and regulate cell cycle progression [9]. It can also regulate the E-cadherin distribution in embryonic lung epithelial branching morphogenesis through MAPK14 and STAT3 [10]. However the role of miR-106b in cancer metastasis remains unclear.

Animal models are widely used in cancer research for studying the development of cancer and metastasis. Microenvironment affects the development of cancer and metastasis [11]. Orthotopic animal model can provide a more relevant model of cancer development in human, and the efficiency for development of metastasis is higher [12]. This study demonstrated the involvement of miR-106b in development of HCC metastasis by orthotopic animal HCC model. Clinicopathological significance of miR-106b was also evaluated.

## Materials and Methods

### Cell Lines and Tumor Specimens

HCC cell lines PLC8024, HepG2, Hep3B, Huh7 (ATCC, Manassas, VA), MHCC97H (obtained from Liver Cancer Institute, Fudan University, Shanghai, China) [13] and immortalized normal hepatocyte cell line MIHA (kind gift from Dr J Roy-Chowdhury, Albert Einstein College of Medicine) [14] were cultured in Dulbecco's Modified Eagle's Medium (DMEM) supplemented with 10% fetal bovine serum (FBS) (Life Technologies, Carlsbad, CA).

HCC clinical samples were collected with informed written consent and approval by the Institutional Review Board of the University of Hong Kong from HCC patients who underwent hepatectomy in Department of Surgery, Queen Mary Hospital, University of Hong Kong Medical Center (Hong Kong SAR, China). Clinicopathological data were collected.

### Orthotopic Implantation Tumor Model and Establishment of Primary and Metastatic Cell Lines

Animal studies were approved by Committee on the Use of Live Animals in Teaching and Research at the University of Hong Kong. HCC cell lines, PLC8024 and MHCC97H cells were labeled with luciferase by lentivirus infection. 5×10^5^ cells were orthotopically implanted into the liver of 4–6 weeks old CB17 SCID mice under anesthesia. Tumor formation was monitored by IVIS100 *in vivo* imaging system (Caliper Life Science, Hopkinton, MA) with intra-peritoneal injection of 3 mg luciferin. After 10–12 weeks of inoculation, mice were sacrificed. Primary liver tumor and metastatic lung nodules were isolated and subjected to primary culture. Stable cell lines were checked by luciferase activity to confirm the cells originated from the parental HCC cell lines.

### Wound-healing Assay and Trans-well Invasion Assay

Cell migration ability was measured by wound-healing assay. Full confluent cells were seeded into 24-wells plate. Acellular area was created by scraping using a pipette tip. Wound closure was measured at 24 and 48 hours interval.

Trans-well invasion assay was performed using matrigel invasion chamber (BD Biosciences, Bedford, MA). 2×10^5^ cells were seeded into the upper chamber with serum-free DMEM. DMEM with 10% FBS were put into lower compartment as chemo-attractant. Cells were allowed to invade for 48 hours. Remaining cells in the upper chamber were scraped out by cotton swap. Matrigel membranes were fixed with ice-cold methanol and stain with 0.1% crystal violet solution. Membranes were then destained and visualized under microscope. Each experiment were performed in triplicate and repeated twice.

### Stress Fiber Formation Analysis by Phalloidin Staining

Cells were cultured on chamber slide for 24 hours and serum-starved for another 24 hours. After 48 hours, cells were fixed with 4% paraformaldehyde and cell membranes were permeabilized with 0.1% TritonX100. Slides were then blocked by 1% BSA, and FITC-conjugated or TRITC-conjugated phalloidin (Sigma, St Louis, MO) was hybridized onto the slide at 37°C for 1 hour. Images were then visualized by fluorescent microscopy.

### Total RNA Isolation and miRNA Microarray Analysis

Total RNAs from cell lines and HCC clinical samples were extracted by Trizol reagent (Life Technologies, Carlsbad, CA) following manufacture's protocol.

miRNA microarray analysis was carried out by NCode miRNA expression profiling service (Life Technologies, Carlsbad, CA). Total RNAs (10 µg) were enriched by PureLink miRNA isolation kit (Life Technologies, Carlsbad, CA). Enriched miRNAs were polyadenylated and subsequently tagged with specific sequences to enable the detection of fluorescents. Tagged miRNAs were then purified and hybridized onto NCode Multi-Species miRNA Microarray V2.0 (Life Technologies, Carlsbad, CA), containing probes for Sanger mirBASE 9.0, overnight at 52°C. Slides were then subjected to stringency wash and hybridized to AlexaFluor3 or AlexaFluor5 at 62°C for 4 hours. Slides were then washed and scan using GenePix4000B microarray scanner (Molecular Device, Sunnyvale, CA). Data were captured and analyzed by GenePix Pro software (Molecular Device, Sunnyvale, CA).

### First-Strand cDNA Synthesis

First-strand cDNA synthesis for miRNA QPCR analysis was performed by TaqMan miRNA Reverse Transcription Kit and MegaPlex Primer Pool (Life Technologies, Carlsbad, CA). Total RNA (350 ng) was subjected to reverse transcription with MegaPlex Primer Pool as RT-primer.

### QPCR Analysis

TaqMan MicroRNA Assays were used for QPCR analysis. Reaction mixture containing 1XTaqMan Universal PCR Master Mix (Life Technologies, Carlsbad, CA), 1XTaqMan MicroRNA Assay and 1∶15 diluted cDNA was subjected to thermal cycling on 7900HT Fast Real-Time PCR System (Life Technologies, Carlsbad, CA). U6 snRNA was used as reference for the expression of the mature miRNAs. The cycling conditions were 95°C 10 min, followed by 40 cycles at 95°C 15 sec and 60°C 1 min. Relative miRNAs expression was calculated by 2^−ddCt^ methods.

### miRNA Knock-down and Over-expression Functional Studies

miRNA-106b LNA knock-down probe and the scramble control (Exiqon, Vedbaek, Denmark) were transfected into the miR-106b over-expressed cell line, PLC-LM, using Lipofectamine 2000 (Life Technologies, Carlsbad, CA) according to manufacturer's protocol.

Pre-miR-106b was cloned into pCDH-CMV-MCS-EF1-copGFP vector (System Biosciences, Mountain View, CA). Pseudoviral particles were prepared by the LentiStarter Kit (System Biosciences, Mountain View, CA) following the manufacturer's protocol. Pseudoviral particles were used for transduction in PLC-PT, Huh7, and Hep3B cell lines. One week after transduction, GFP+ cells were sorted using MoFlow cell sorting system (Beckman Coulter Inc., Brea, CA). GFP+ cells were harvested and QPCR analysis was employed to confirm the over-expression of miR-106b.

### Western blotting for RhoGTPases and EMT markers

Protein lysate were obtained from cell lines using RIPA buffer (Cell Signaling Technology, Danvers, MA). RhoGTPases, RhoA and RhoC (Cell Signaling Technology, Danvers, MA), and EMT markers, E-cadherin, N-cadherin (Cell Signaling Technology, Danvers, MA), Vimentin (Abcam Inc., Cambridge, MA), and TWIST1 (Sigma, St Louis, MO), were immune-blotted as previously described [15].

### Statistical Analysis

Statistical analysis was performed by SPSS16.0. Continuous data and categorical data were analyzed by Student's t-test and chi-square test respectively. P-value<0.05 was consider as statistically significant.

## Results

### Establishment of Primary and Metastatic Tumor Cell Lines

**Figure 1:**
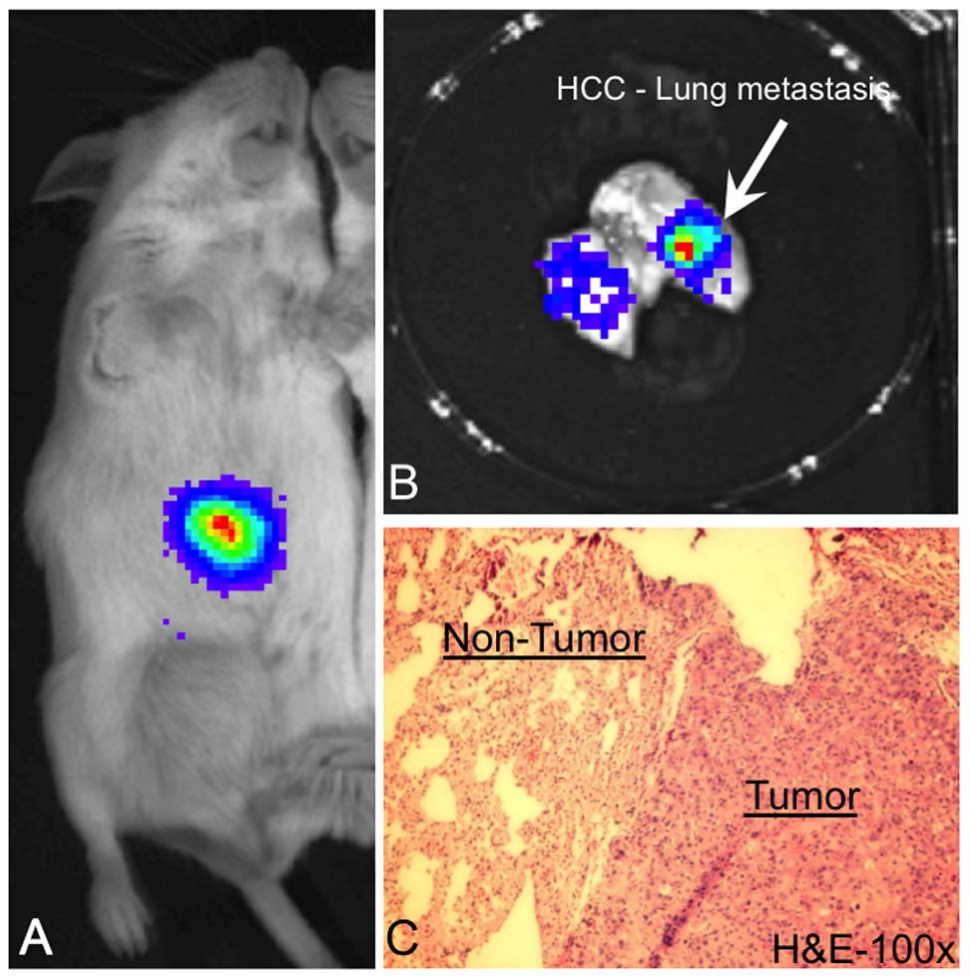
Orthotopic HCC metastasis animal model. (A) Primary liver tumor was detected after 1-week orthotopic implantation using luciferase labeled HCC cell line, PLC8024. (B) Metastatic nodules were formed in the lung of the SCID mice at week 10. (C) H&E staining for the lung of the SCID mice show that luciferin signal from the lung nodules are orginated from the tumor cells.

HCC cell lines PLC8024 and MHCC97H, labeled with luciferase, were orthotopically implanted into liver of SCID mice (Fig. 1A). After 10–12, mice were sacrificed and lung metastatic nodules were discovered in mice (Fig. 1B). Another mouse was sacrificed for H&E staining to confirm the luciferase signal originated from the metastatic lung lesions (Fig. 1C). Tumor cells were isolated for primary culture from both primary liver tumors and metastatic lung tumors. Stable cell lines derived from PLC8024 cell line, namely PLC-PT (Primary Tumor) and PLC-LM (Lung Metastasis), and cell lines from MHCC97H, namely MHCC97H-PT and MHCC97H-LM, were established. All derived cell lines show positive luciferase activity, indicating that all isolated cells originated from the parental luciferase-labeled cell lines. No observable morphological difference was identified in these cell lines (Data not shown).

### In vitro and in vivo Functional Characterization of the Primary and Metastatic Cell Lines

Cell proliferation rates of both primary and metastatic cell lines were analyzed by MTT assay. No obvious differences between primary tumor and lung metastasis cell lines for both PLC8024 and MHCC97H derived cell lines were observed (Data not shown).

**Figure 2:**
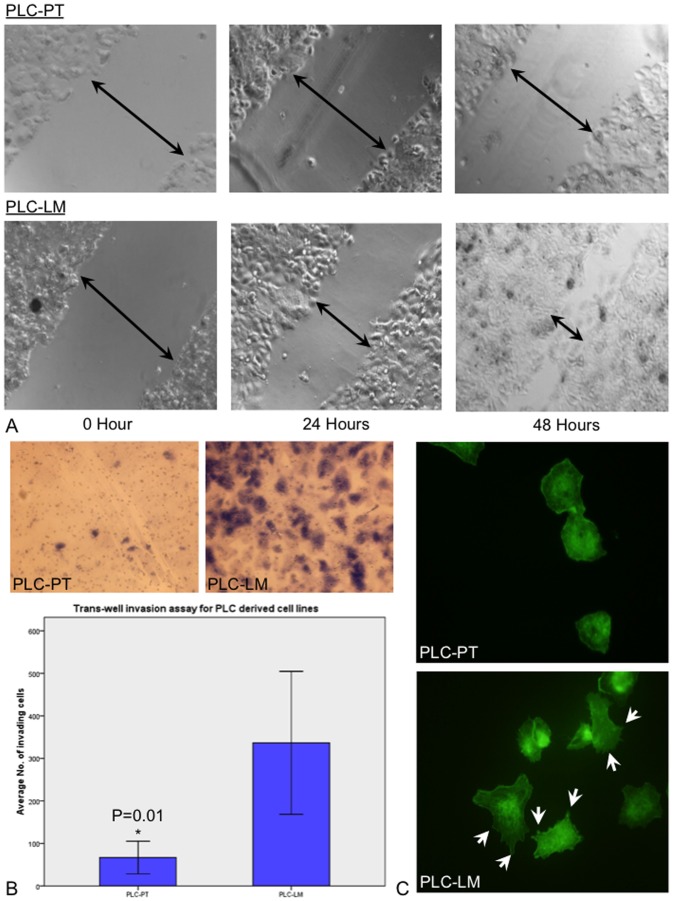
*In vitro* functional studies for PLC-PT and PLC-LM cell lines. (A) Cell migration ability were compared by wound healing assay. Wound closure was observed in the PLC-LM but not in the PLC-PT at 24 and 48 hours. (B) Trans-well invasion assay for PLC-PT and PLC-LM. The number of invading cells in PLC-LM are significantly higher than the PLC-PT (p = 0.01). (C) Phalloidin staining for PLC-PT and PLC-LM. Stress fiber (white arrow) was observed in PLC-LM but absent in PLC-PT.

Migration ability was measured by wound-healing assay. Fig. 2A shows the wound closure after 24 and 48 hours for PLC-PT and PLC-LM cell lines. Wound closure was observed in PLC-LM at 24 and 48 hours but not in PLC-PT cell line. Similar experiment was performed in MHCC97H-PT and MHCC97H-LM cell lines. No obvious difference between MHCC97H primary and the metastatic cell lines was observed (Data not shown).

Cell invasion ability was measured by trans-well invasion assay (Fig. 2B). The invasion ability of PLC-LM was higher than PLC-PT cell line, and the number of invading cells in PLC-LM was significantly higher than in PLC-PT (p = 0.01). No obvious difference was observed in MHCC97H derived cell lines (Data not shown).

Metastatic ability of the PLC-PT and PLC-LM cell lines were compared by using the orthotopic animal model (Data not shown). The PLC-PT cell shows a lower metastatic ability compared to the parental PLC8024 and PLC-LM after 10 weeks of inoculation. For the PLC-LM, it has the highest metastatic ability amount three cell lines and most of the mice develop lung metastasis at week 10.

### Cytoskeleton Rearrangement Promotes Cell Migration and Cell Invasion

Cytoskeleton structure was visualized by phalloidin staining (Fig. 2C). PLC-LM cell line had a well-established cytoskeleton network with stress fiber protruding out from the cell surface. These features were absent in the PLC-PT cell line.

### MicroRNA Microarray Profiling

The primary tumor and lung metastasis cell lines of the two sets of phenotypically similar cell lines (PLC-PT vs PLC-LM and MHCC97H-PT vs MHCC97H-LM) were compared by NCode miRNA microarray profiling service. Array data were deposited at the Gene Expression Omnibus (GEO accession no.: GSE43445). MicroRNA candidates were selected by >2-fold difference in expression with at least 4 out of 6 spots were statistically significant (p-value<0.001). With criteria mentioned above, 67 miRNAs candidates were identified from PLC8024 derived cell lines, whereas 30 candidates were selected from MHCC97H derived cell lines. Fifteen common candidate human miRNAs were identified from the microarray analysis from these two sets of cell line (Table 1).

**Table 1:** Candidate miRNAs identified from miRNA microarray analysis.

Candidate miRNAs	Fold-difference by microarray	
	PLC8024 derived cell lines	MHCC97H dervied cell lines
hsa-miR-21	16.53	10.26
hsa-miR-26b	11.94	3.19
hsa-miR-29a	8.72	2.15
hsa-miR-20a	8.38	3.47
hsa-miR-27a	7.23	2.67
hsa-miR-19b	7.06	2.96
hsa-miR-16	6.91	2.55
hsa-miR-106b	6.21	2.63
hsa-miR-106a	5.75	1.98
hsa-let-7a	5.16	2.28
hsa-miR-27b	4.99	3.27
hsa-miR-18a	4.55	2.24
hsa-let-7f	4.06	3.83
hsa-miR-15a	2.86	2.74
hsa-miR-195	2.24	2.00

### Quantitative PCR (QPCR) Analysis in HCC Cell Lines and Clinical Samples

To validate microarray results, PLC8024 and MHCC97H derived cell lines were subjected to QPCR analysis. miR-106b expression in other HCC cell lines was also compared. All HCC cell lines show higher expression of miR-106b than normal hepatocyte cell line, MIHA (Fig. S1A). Furthermore, two metastatic cell lines had higher miR-106b expression than the corresponding primary tumor cell lines (Fig. S1B).

Target miRNAs, which were associated with cell migration capacity *in vitro* and metastatic ability *in vivo*, were identified by microarray analysis. The clinical relevance of top 8 over-expressing miRNAs from microarray analysis was first analyzed in 20 pairs of matched HCC tumor and non-tumor sample. Amount these miRNAs, miR-21 and miR-106b show clinical significance in this 20 HCC clinical sample cohort. This two miRNA targets were further analyzed by QPCR in another 79 pairs HCC sample (Total 99 pairs). miR-21 and miR-106b showed significant up-regulation in tumors compared with the non-tumor tissues (p = 0.0059 and 0.0005 respectively) (Fig. S1C and S1D). We correlate the expression of the two miRNAs with clinicopathological data (Table 2). The only significant correlation observed was higher tumor grade in tumors with miR-106b over-expression (T vs NT >2-fold) (p = 0.014). There was also a trend towards advanced TNM stage with miR-106b over-expression, but the difference was marginally not significant (p = 0.061).

**Table 2:** Clinicopathological analysis for miR-21 and miR-106b expression in HCC clinical samples.

Clinicopathological parameters		n	p-value 1	
		(Total = 99)	miR-106b	miR-21
Sex:	Male	81	0.667	0.165
	Female	18		
Age:	<60	62	0.385	0.770
	> = 60	37		
Tumor Grade:	Well/Moderate	70	**0.014**	0.988
	Poor	12		
TNM Staging:	Stage I/II	36	0.061	0.652
	Stage III/IV	62		
Microvascular Invasion:	+ve	47	0.243	0.890
	−ve	51		
Tumor Size:	<5 cm	27	0.267	0.553
	> = 5 cm	71		

1 *Chi-square test*.

### In vitro Functional Studies of miR-106b Konck-down or Over-expressed Cell Lines

**Figure 3:**
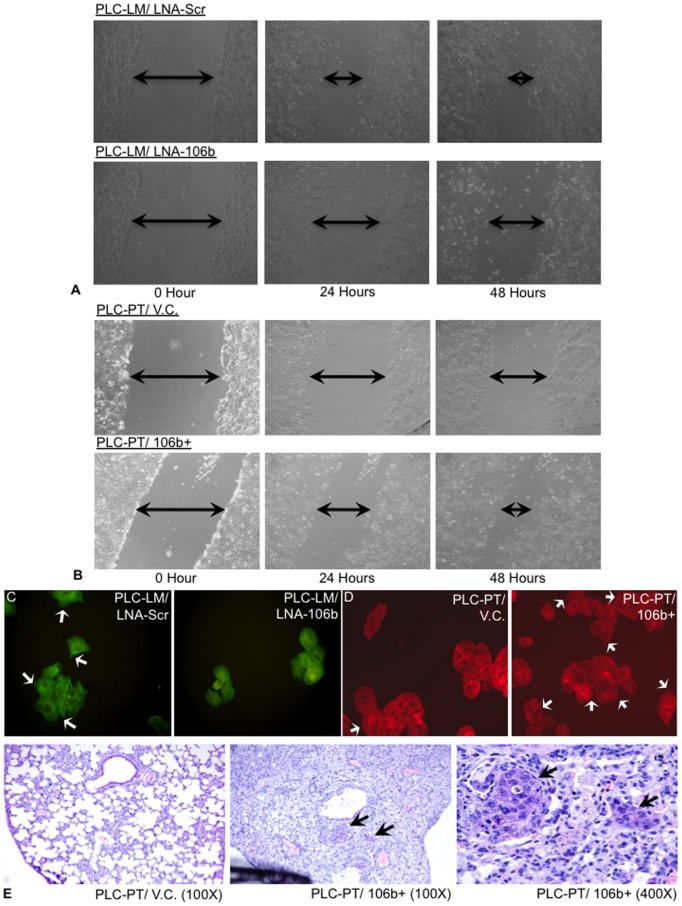
*In vitro* and *in vivo* studies for miR-106b. miR-106b was over-expressed in PLC-PT and knock-down in PLC-LM cells. (A) Wound-healing assay for miR-106b knock-down using PLC-LM cells and (B) miR-106b over-expression using PLC-PT cells. Higher miR-106b expressing cells, PLC-LM/Scr and PLC-PT/106b+, can promote cell migration *in vitro*. (C) Stress fiber (white arrows) protruding out from cell surface in high miR-106b expressing cells, PLC-LM/LNA-Scr, but not in the miR-106b knock-down cells, PLC-LM/LNA-106b. (D) Similar founding was observed in miR-106b over-expressing experiment. (E) H&E staining for *in vivo* studies showing lung metastatic nodules were present in miR-106b over-expressing cell, PLC-PT/106b+.

QPCR analysis showed successful miR-106b knock-down in PLC-LM cells by Locked Nucleic Acid (LNA) with >50% efficiency from Day 1 to Day 4 after transfection (Fig. S1E), while the lentiviral system stably over-expressed 9-fold higher miR-106b than the empty vector control in PLC-PT cells (Fig. S1F). Other 2 HCC cell lines, Huh7 and Hep3B, with lentiviral transduction also show around 6-fold over-expression of miR-106b compare to the corresponding vector control (Fig. S1F). MTT assay and trans-well invasion assay showed that miR-106b expression level affected neither the cell proliferation rate nor the invasion ability (Data not shown). However, cell lines with higher miR-106b expression, PLC-LM/LNA-Scr and PLC-PT/106b+, had higher migration ability. Fig. 3A and 3B showed the effect on wound healing assay after miR-106b LNA knock-down in PLC-LM cell line and miR-106b over-expression in PLC-PT cell line respectively. Since stress fiber formation can promote cell migration ability, we further analyzed the stress fiber formation in these cell lines by phalloidin staining (Fig. 3C and 3D). The results demonstrated that miR-106b expression can promote formation of stress fiber, hence, increase migration ability of the HCC cells.

**Figure 4:**
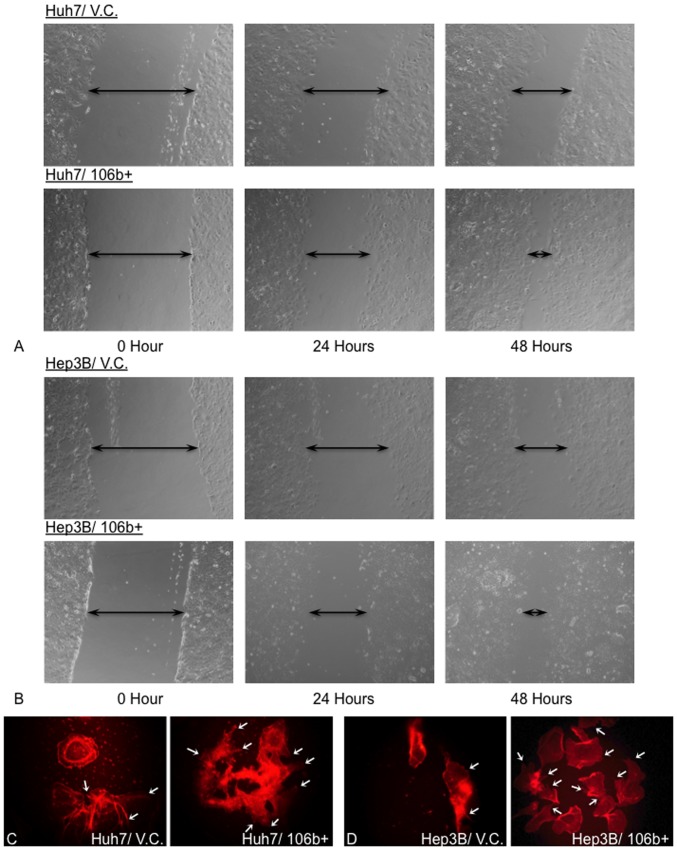
*In vitro* functional studies for miR-106b in Huh7 and Hep3B cell lines. Cell migration ability was compared in Huh7 (A) and Hep3B (B) cell lines with or without over-expression of miR-106b. miR-106b over-expressing cells, Huh7/106b+ and Hep3B/106b+, show enhanced cell migration ability when compare to its corresponding vector control, Huh7/V.C. and Hep3B/V.C., at 24 and 48 hours interval. Stress fiber formation was visualized by phalloidin staining (C & D). More stress fiber (white arrow) was observed in miR-106b over-expression cells in Huh7/106b+ (C) and Hep3B/106b+ (D) cell lines compare to the vector control cell lines, Huh7/V.C. and Hep3B/V.C.

To confirm the functional role of miR-106b in other HCC cell lines, wound healing assay and phalloidin staining were performed in other 2 HCC cell lines, Huh7 and Hep3B. The wound healing assay showed that miR-106b over-expression can enhance the cell migration ability in vitro in both cell lines (Fig. 4A and 4B). In addition, the phalloidin staining demonstrated that both cell lines process more stress fiber in the miR-106b over-expressing cells, but not in the vector control (Fig. 4C and 4D).

### Orthotopic Metastasis Animal Model for PLC-PT with miR-106b Over-expression

PLC-PT cells stably over-expressing miR-106b were implanted into liver of SCID mice. Mice were scarified at week 10, lungs were analyzed by H&E staining (Fig. 3E). Lung metastatic nodules were observed in 4 out of 5 mice (80%) in the miR-106b over-expressing group, whereas in the empty vector control group, only 1 out of 5 mice (20%) developed lung metastasis. This result demonstrated that miR-106b enhanced metastatic capacity of the PLC-PT cells.

### miR-106b Over-expression Up-regulated RhoGTPases Expression

**Figure 5:**
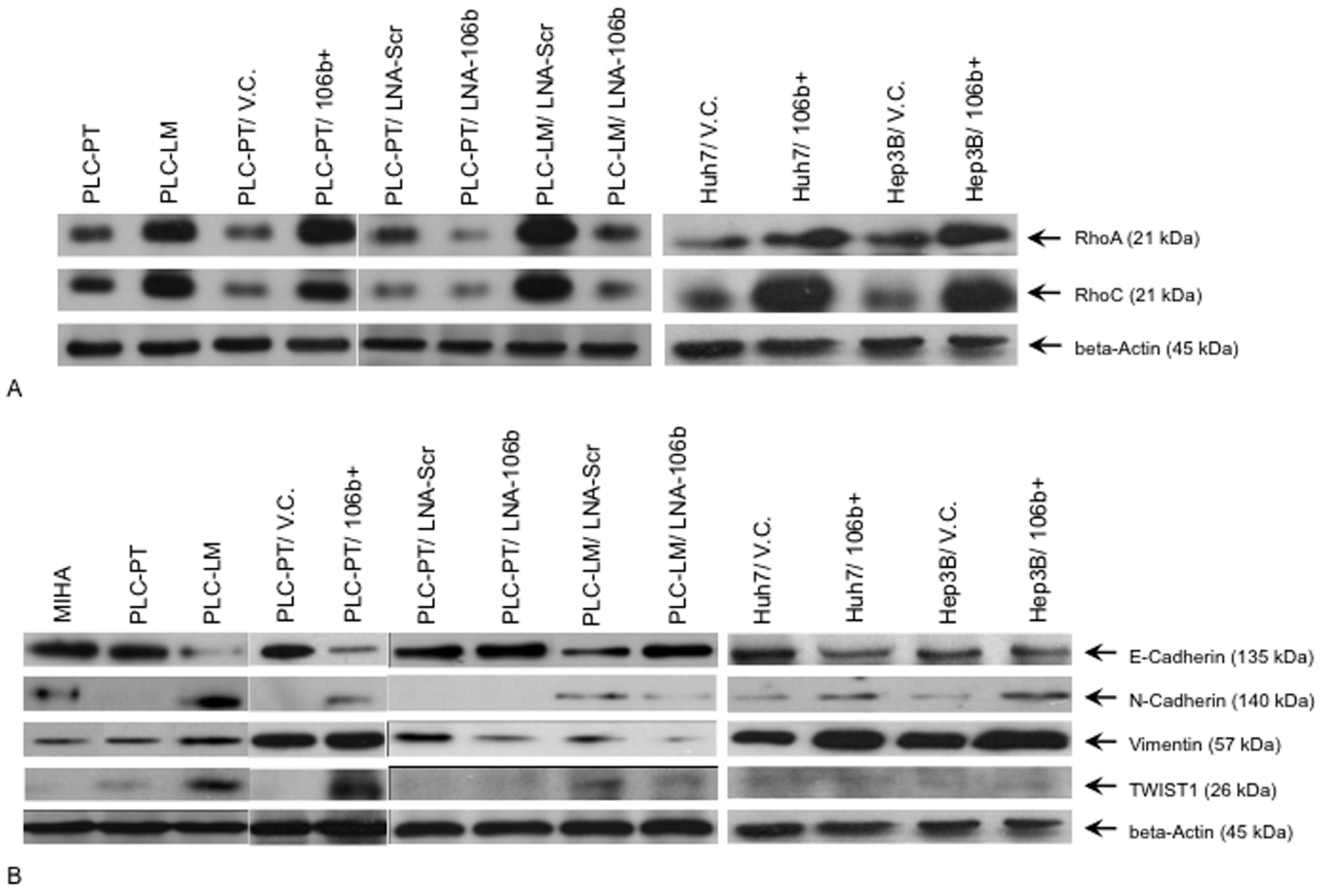
Alteration of RhoGTPases and EMT markers expression attributed to miR-106b expression. (A) High miR-106b expressing cells (PLC-LM, PLC-PT/106b+, PLC-PT/LNA-Scr, PLC-LM/LNA-Scr, Huh7/106b+, and Hep3B/106b+) show over-expression of RhoA and RhoC by western blotting. (B) Expression of EMT markers, E-cadherin, N-cadherin, Vimentin, and TWIST1, were analyzed by western blot. PLC-LM shows higher expression of N-cadherin, Vimentin, and TWIST1, but less E-cadherin, indicating EMT process is activated in the metastatic cell line. miR-106b knock-down cells decrease mesenchymal markers expression and increase epithelial marker expression indicating miR-106b expression can activate EMT process. N-cadherin and TWIST1 expression are too low to be detected in PLC-PT. EMT activation was also observed in miR-106b over-expressing cell lines, Huh7/106b+ and Hep3b/106b+, but not in its corresponding vector control.

Expression of RhoGTPases, RhoA and RhoC, were analyzed by western blot (Fig. 5A). Over-expression of RhoGTPases can be observed in miR-106b over-expressing cell lines, PLC-LM, PLC-PT/106b+, PLC-PT/LNA-Scr, PLC-LM/LNA-Scr, Huh7/106b+, and Hep3B/106b+, but not in the corresponding control. Our result suggested that miR-106b over-expression can up-regulate RhoA and RhoC expression.

### Regulation of Epithelial-mesenchymal Transition Process by miR-106b

miR-106b were transiently knocked down by LNA in both PLC-PT and PLC-LM cell lines. Four EMT markers were analyzed by western blot. Epithelial marker E-cadherin, mesenchymal markers N-cadherin, TWIST1, and Vimentin were analyzed. Our results demonstrated that upon miR-106b knock-down, the expression of epithelial marker increased while the mesenchymal markers decreased (Fig. 5B). This result suggested that miR-106b over-expression can activate EMT process and promote cell migration *in vitro* and metastasis *in vivo*. Meanwhile, cells transduced with lentivirus over-expressing miR-106b (PLC-PT/106b+, Huh7/106b+, Hep3B/106b+) also show activation of EMT compare to their corresponding vector control (PLC-PT/V.C., Huh7/V.C., Hep3B/V.C.) (Fig. 5B).

## Discussion

HCC is a cancer prone to vascular invasion and metastasis, and most patients die from metastatic disease. Understanding the biology of metastasis in HCC is critical to improve patient prognosis. As primary treatment for metastatic HCC is usually not by surgery, it is difficult to collect metastatic tumor specimens from patients. In this study, orthotopic metastasis HCC animal model is used to study the role of microRNAs in development of metastasis in HCC.

The primary tumor and lung metastatic cell lines derived from PLC8024 showed different invasiveness *in vitro*. The PLC-LM cell line was highly invasive and had higher migration ability. However, the proliferation rates are similar in PLC-PT and PLC-LM cell line. This suggested that metastasis development *in vivo* is related to enhanced cell migration and invasion ability rather than cell proliferation. In contrast, we did not observe higher migration capacity in MHCC97H-LM when compared to the MHCC97H-PT cell line. This may be due to the facts that MHCC97H is already a highly metastatic cell line, some of the molecular pathways involved in promoting the aggressive phenotype are deregulated, so the difference between MHCC97H-LM and MHCC97H-PT were not obvious. Even we can observed miR-106b expression is higher in MHCC97H-LM, this does not make any differences in the *in vitro* functional characteristics of this two cell lines since some of the down-stream molecule are deregulated due to the aggressive nature in the parental cell line, MHCC97H.

To explore the role of miRNAs in development of metastasis, two metastatic cell lines derived from PLC8024 and MHCC97H were compared with the corresponding primary tumor cell lines by microarray analysis. More miRNAs were differentially expressed in the PLC8024 derived cell lines. These genotypic differences may contribute to the phenotypic differences *in vitro* between the primary tumor and lung metastasis cell lines. The metastatic cell line showed higher migration and invasion abilities than the primary tumor cell line in PLC8024 derived cell lines, but not in the MHCC97H derived cell lines. However, metastatic ability *in vivo* was similar between PLC8024 and MHCC97H cell lines and those commonly differential expressed miRNAs maybe related to the development of metastasis. By comparing miRNA microarray profile of the PLC8024 and MHCC97H derived cell lines, 15 human miRNAs were differentially expressed in primary tumor cell lines versus the metastatic cell lines. Although our focus in this study was miR-106b, the highest differentially expressed miRNA was miR-21, which is a well-known miRNA involved in various kinds of cancer including HCC [3], [16], [17] and has been proven to promote metastasis in breast and colorectal cancer [18], [19]. In contrast, miR-106b is a new candidate miRNA with potential importance in HCC metastasis for further *in vitro* and *in vivo* studies.

QPCR analysis for miR-21 and miR-106b expression in HCC and nontumorous liver was performed. U6 was chosen as the reference in our studies as it is stably expressed in all cell lines and clinical sample cohort. Several studies have demonstrated the critical role of miR-21 in HCC carcinogenesis [3], [16], [20], [21] and our orthotopic model confirmed its functional role in HCC progression. We further confirm the clinical significance of miR-21 by showing its over-expression in HCC tumor. We also analyzed the clinical relevance of miR-106b expression. Our results demonstrated that miR-106b was over-expressed in tumor tissue compared with the adjacent non-tumor tissue. We correlated clinicopathological parameters of the patients with miR-106b expression and observed that over-expression of miR-106b (>2-fold difference between tumor and non-tumor) correlated with higher tumor grade, which is associated with more aggressive disease and poor prognosis. Moreover, there was higher expression of miR-106b in more advanced tumor stage, although the difference was marginally not significant.

To investigate the functional role of miR-106b, we knocked down miR-106b in the lung metastasis cell line with high miR-106b expression and over-expressed miR-106b in the primary tumor cell lines with low miR-106b expression. LNA knock-down *in vitro* were transient and >50% knock-down could be attained within 4 days post-transfection. We performed a series of experiments including MTT assay, migration and invasion assay to study the functions of miR-106b. Our results suggested that migration ability decreased upon miR-106b knock-down without affecting cell proliferation rate and invasion ability, suggesting the main functional role of miR-106b in enhancing cell motility. To confirm this finding, we also over-expressed miR-106b in PLC-PT cell line. Migration ability was increased when we over-expressed miR-106b in the cells without affecting cell proliferation rate and invasion ability. Since miR-106b could increase cell migration ability in PLC-PT cell lines, we visualized the stress fiber formation, which can promote cell migration, by phalloidin staining. In order to confirm our finding in other HCC cell lines, we perform both wound healing assay and phalloidin staining in Huh7 and Hep3B cell lines. We observed higher cell migration ability with increase in stress fiber formation upon miR-106b over-expression. We demonstrated that over-expression of miR-106b can increase stress fiber formation, hence, enhancing migration ability *in vitro*.

PLC-PT cell line with stable over-expression of miR-106b was orthotopically implanted into the mice to examine the *in vivo* metastatic potential. We observed lung metastatic nodules in 80% of mice inoculated with miR-106b over-expressing cells, whereas only 20% mice developed lung metastasis in the empty vector control group. This showed that miR-106b expression could promote formation of lung metastatic nodules in mice, further corroborating a critical role of miR-106b in development of HCC metastasis.

RhoGTPases, RhoA and RhoC, are reported to be involved in regulation of actin polymerization and organization of actin cytoskeleton resulting in stress fiber formation [22]. Our *in vitro* studies demonstrated miR-106b over-expression can increase cancer cell motility by inducing stress fiber formation. Since RhoA and RhoC are essential RhoGTPases involved in stress fiber formation and cell migration, expression level of RhoA and RhoC were analyzed. Our results demonstrated that miR-106b over-expressing cells, PLC-LM, have higher expression level for RhoA and RhoC than the PLC-PT. Knock-down of miR-106b significantly down-regulate RhoA and RhoC expression, but not in the scramble control. This indicated that expression of miR-106b can indirectly regulate the expression of RhoGTPases. This observation can be confirmed by up-regulation of RhoGTPases in miR-106b over-expressing transfectant, PLC-PT/106b+, Huh7/106b+, and Hep3b/106b+. Up-regulation of RhoA and RhoC are essential for cell migration and cancer metastasis in various cancers [23]. Our result suggested that increase of cell motility *in vitro* and metastasis development *in vivo* were affected by the indirect regulation of RhoA and RhoC through miR-106b regulation.

Previous studies have already demonstrated that miR-106b was involved in various important signaling pathways such as PTEN tumor-suppressive pathway [24], and TGF-beta signaling pathway [25]. It is also involved in cell cycle progression through p21 [26] and RB [27]. We postulated that miR-106b over-expression may regulate EMT process in cancer. Our *in vitro* and *in vivo* data supported this hypothesis. With higher miR-106b expression, the cells exhibited higher metastatic potential. When miR-106b expression was down-regulated, expression of epithelial marker, E-cadherin, increased while mesenchymal markers, N-cadherin, Vimentin, and TWIST1, decreased. Taken together, our data suggest a role of miR-106b in metastasis of HCC via activation of EMT process and enhancement of cell motility. This is the first study reporting the role of miR-106b in HCC metastasis and implies a potential strategy to inhibit metastasis in HCC by targeting miR-106b.

## Supporting Information

Figure S1 **QPCR analysis in cell lines and clinical sample.** (A) miR-106b expression in HCC cell lines is higher than immortalized normal hepatocyte cell line MIHA. (B) Higher miR-106b expression was observed in metastatic cell lines than the primary tumor cell lines. (C) miR-21 and (D) miR-106b expression were analyzed in HCC clinical sample by QPCR. Expression of miR-21 and miR-106b was significantly higher in HCC tumor tissue. (E) Successful knock-down of miR-106b expression was attained within 4 days after transfection. (F) Over-expression of miR-106b in PLC-PT, Huh7 and Hep3B cell lines can be attained by lentiviral transduction by comparing with the empty vector control.
(TIF)
