# Supplementary figures and images for: Over-Expression of miR-106b Promotes Cell Migration and Metastasis in Hepatocellular Carcinoma by Activating Epithelial-Mesenchymal Transition Process
Source: PLoS One. 2013 Mar 6;8(3):e57882. doi: 10.1371/journal.pone.0057882 (PMC3590302; doi:10.1371/journal.pone.0057882)

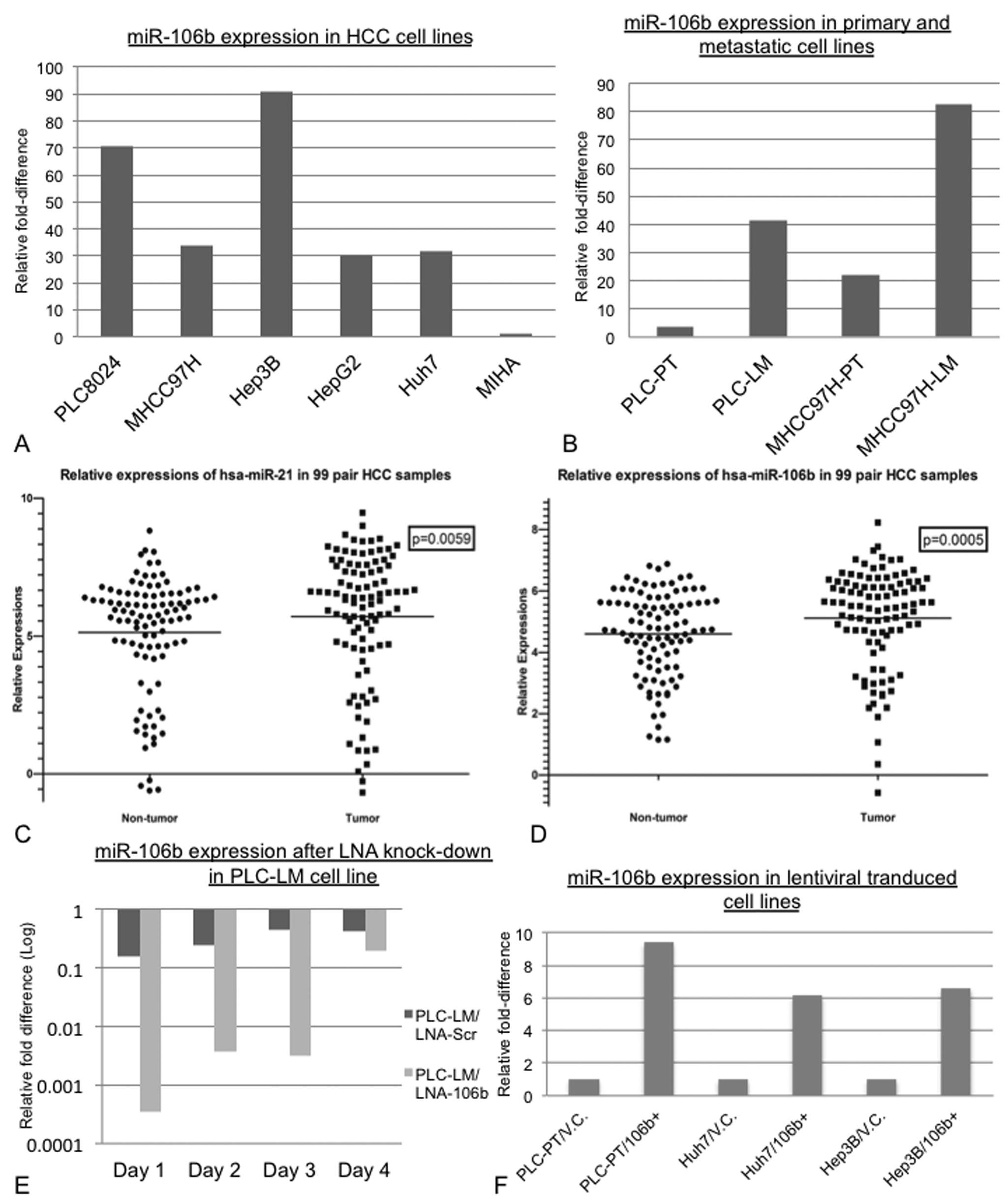

Supplement: Figure S1 — QPCR analysis in cell lines and clinical sample. (A) miR-106b expression in HCC cell lines is higher than immortalized normal hepatocyte cell line MIHA. (B) Higher miR-106b expression was observed in metastatic cell lines than the primary tumor cell lines. (C) miR-21 and (D) miR-106b expression were analyzed in HCC clinical sample by QPCR. Expression of miR-21 and miR-106b was significantly higher in HCC tumor tissue. (E) Successful knock-down of miR-106b expression was attained within 4 days after transfection. (F) Over-expression of miR-106b in PLC-PT, Huh7 and Hep3B cell lines can be attained by lentiviral transduction by comparing with the empty vector control. (TIF) [file pone.0057882.s001.tif]
